# Using infectious intestinal disease surveillance data to explore illness aetiology; a cryptosporidiosis case study

**DOI:** 10.1016/j.healthplace.2008.06.005

**Published:** 2009-03

**Authors:** Iain R. Lake, Gordon Nichols, Florence C.D. Harrison, Graham Bentham, R. Sari Kovats, Chris Grundy, Paul R. Hunter

**Affiliations:** a School of Environmental Sciences, University of East Anglia, Norwich NR4 7TJ, UK; b Centre for Infections, Health Protection Agency, 61 Colindale Avenue, London, UK; c School of Health Policy and Practice, University of East Anglia, Norwich, UK; d Public and Environmental Health Research Unit, London School of Hygiene and Tropical Medicine, London, UK

**Keywords:** Surveillance data, GIS, Crypsorpodisiodis, Environment, Water supply

## Abstract

Infectious intestinal disease (IID) surveillance data are an under-utilised information source on illness geography. This paper uses a case study of cryptosporidiosis in England and Wales to demonstrate how these data can be converted into area-based rates and the factors underlying illness geography investigated. Ascertainment bias is common in surveillance datasets, and we develop techniques to investigate and control this. Rural areas, locations with many livestock and localities with poor water treatment had elevated levels of cryptosporidiosis. These findings accord with previous research validating the techniques developed. Their use in future studies investigating IID geography is therefore recommended.

## Introduction

Worldwide infectious intestinal disease (IID) is an important health concern. In the USA there are estimated to be 73 million cases annually, implying that over 25% of the population are affected. This leads to over 325,000 hospital admissions and 5000 deaths (Mead et al., 1999). Similar rates are reported in England and Wales (Adak et al., 2002). In many countries, the main source of information on how IID incidence varies geographically are national surveillance systems. These collect data from a number of sources including the voluntary reporting of organisms identified by public health and other diagnostic microbiology laboratories, and reports of general outbreaks of IID (Wheeler et al., 1999). England and Wales, Sweden and the United States are examples of countries with such surveillance systems (Stanwell-Smith et al., 2003). The health data in these systems are commonly used for investigating trends in IID incidence and identifying outbreaks.

However, little attention has been paid to the potential for these data to map illness geography and investigate the factors underlying these patterns. There are two main reasons for this under-utilisation. The first is the practical difficulty of linking health surveillance data to geographical information on aetiologically plausible factors. This is because, for reasons of confidentiality, the data rarely contain the address of the affected individual. The second is ascertainment bias in IID surveillance datasets because, as an example, not all people infected with an IID visit their doctor. This could cause any geographical analysis of disease rates to produce misleading results if ascertainment and a potential explanatory variable are correlated. This research question has received little attention (Sethi et al., 1999).

In this paper we use a case study of cryptosporidiosis in England and Wales to explore the usefulness of IID surveillance data for exploring the factors underlying disease geography. This approach is important because our understanding of IID aetiology is largely based upon outbreak investigations which have used questionnaires to examine the importance of proximal risk factors (patient activity) for illness. A thorough examination of the factors underlying IID geography may provide further clues to its aetiology (English, 1996) and such an approach has the added ability to examine the importance of contextual factors, such as the environment within which the infection occurred.

The aims of this paper were:

1. Produce techniques to link IID surveillance data with area based information on socio-economic characteristics, land-use types and water supply quality.
2. Explore the role of these factors in accounting for the geography of cryptosporidiosis across England and Wales.
3. Develop methods to ensure that the results are robust to issues of ascertainment bias in the outcome data.

## Background

### Cryptosporidiosis

Cryptosporidiosis is a severe gastro-enteritis which typically lasts from several days to several weeks. There are no recognised treatments and the illness can be fatal in immune-compromised individuals. The disease is widespread in many developed and developing countries (Hunter, 2003). In England and Wales there are approximately 42,000 cases per annum (Adak et al., 2002).

Cryptosporidiosis is caused by infection with protozoan parasites of the genus *Cryptosporidium*. Infection occurs via the oral route, and the most important species for human illness are *C. hominis* and *C. parvum* (Fayer et al., 2000). In England and Wales there is a large environmental reservoir of *C. parvum* in livestock and transmission is related to direct (e.g. through farm visits (Goh et al., 2004)) or indirect contact (e.g. through contaminated drinking water (Hunter et al., 2004)) with these animals. Humans are the only major environmental reservoir for *C. hominis*, and infection is acquired through direct or indirect contact with other infected individuals (Hunter et al., 2004). Foreign travel is a risk factor for both species (McLaughlin et al., 2000). Once a primary infection has occurred person-to-person transmission may lead to secondary infections.

### Cryptosporidiosis surveillance data in England and Wales

The main method to identify whether an individual has cryptosporidiosis is through a laboratory examination of their stool specimen. The reporting of positive specimens to a national surveillance centre forms the basis of cryptosporidiosis surveillance data in most countries. In England and Wales these data are collated by the Health Protection Agency, Centre for Infections, and contain additional information on the specimen date, the patient age and sex, the laboratory at which the sample was analysed and whether the individual had reported recent foreign travel. The data is not routinely split into *C. hominis* and *C. parvum* as these require molecular methods.

### Ascertainment bias in surveillance data

**Fig. 1:**
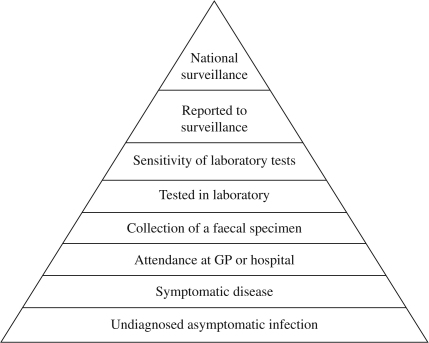
The surveillance pyramid.

Two sources of ascertainment bias need to be considered when using national surveillance data on cryptosporidiosis and other IID. The first is that the reported cases represent a fraction of the total cases in the community. This is due to the reporting pyramid presented in Fig. 1 (Wheeler et al., 1999). Not all individuals with an IID will have symptoms and even symptomatic individuals may not visit their doctor. At the doctor not all patients will have a stool sample taken and not all laboratories will test this sample for cryptosporidiosis and other IID. In England and Wales for every positive cryptosporidiosis case reported to national surveillance there are estimated to be 7.4 cases in the community (Adak et al., 2002).

This leads to under-ascertainment of cryptosporidiosis and other IID in national surveillance datasets. This implies that any study investigating cryptosporidiosis geography will be based upon a fraction of the total cases. This will bias any such study to the null hypothesis due to larger standard errors around the estimates produced. Of greater importance is whether the proportion of omitted cases varies geographically and crucially whether these omissions are correlated with a potential explanatory variable. This may lead to biased coefficient estimates (Sethi et al., 1999). However, there is little evidence on how ascertainment varies geographically. A few studies have looked at ascertainment differences between rural and urban areas but the overall impacts of these are inconclusive. Individuals living in an urban area are more likely to attend general practice (Farmer et al., 2006), but doctors in urban areas are less likely to diagnose an IID (Sethi et al., 1999). Focussing upon the role of the laboratory in ascertainment, laboratories in different parts of England and Wales use varying protocols to decide which stool samples to screen for cryptosporidiosis (Chalmers et al., 2002).

The second source of ascertainment bias is missing attribute information associated with each case. In the case of cryptosporidiosis this is most prevalent in the recording of recent foreign travel by the individual, implying that the infection may have been acquired abroad. In England and Wales national surveillance data record 4% of cryptosporidiosis cases as reporting recent foreign travel (Lake et al., 2005). However, another study recorded this percentage to be nearer 24% (Lake et al., 2007). A study investigating the geography of IID is likely to exclude cases where recent foreign travel has been reported. The poor labelling of foreign travel in surveillance datasets implies that any study on indigenous cases is likely to include cases where the individual has recently travelled abroad.

## Methods

### Cryptosporidiosis data

All cases of cryptosporidiosis reported to national surveillance between 1989 and 2003 were obtained. Any reporting recent foreign travel were removed as the infection may have been acquired abroad. The only information known about case location was the laboratory to which the sample was sent for analysis and there are approximately 250 of these in England and Wales.

A method was developed to assign the cryptosporidiosis counts to different geographical areas. For each laboratory the periods when it was open and closed were determined by assuming that a laboratory was closed if it reported no *Salmonella* or *Campylobacter* for a 3-month period. These infections were chosen because they have a greater incidence than cryptosporidiosis and every laboratory tests all stool samples for these. Between 1989 and 2003 there were 16 time periods when different sets of laboratories were open. A laboratory identified as closed does not necessarily mean that the laboratory was no longer testing stool samples, but would include times when the laboratory failed to report their results to national surveillance. For each of these periods, service areas were calculated for each laboratory which represents localities assumed to be contributing samples to the laboratory. These were estimated by calculating the road distance from each laboratory to each census unit (enumeration district before 1996 and output area after and including 1996) in a GIS and assuming that each unit contributes samples to its nearest laboratory for analysis. The service areas of any laboratories within 10 km of each other were subsequently combined as samples are likely to be sent to either for analysis. This reduced the number of service areas by around 100, the main effect being in London where the service areas from 50 individual laboratories were combined. Each service area comprised a number of undivided census units, an advantageous structure when possible explanatory variables were linked to the health outcome data. This procedure created approximately 135 service areas with an average size of 1142 km^2^ but ranging in size from 106 to 3770 km^2^. Because the open laboratories changed over time there were 16 different service area configurations between 1989 and 2003. The method was validated against a dataset of 3500 cryptosporidiosis cases where the laboratory that the sample was sent to for analysis was known, and where detailed information (postcode) was available on where the affected individual lived. This indicated that 87% of cases were assigned to the correct laboratory validating our method.

**Fig. 2:**
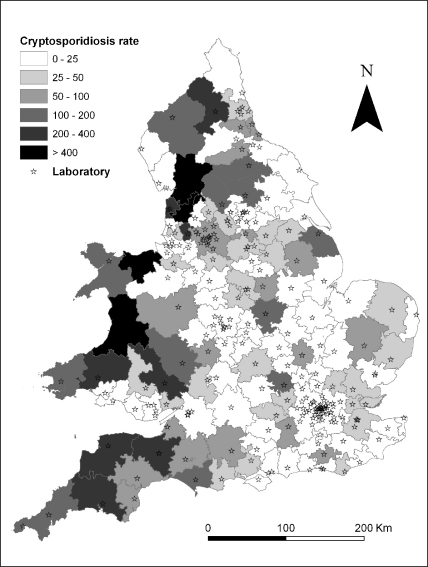
Laboratory service areas and cryptosporidiosis rates in 0–4 year old (per million population); March–June 2000.

For each service area the age structure of the population was obtained from the UK census and used to construct an all age standardised incidence ratio (SIR; calculated using indirect standardisation) for each period. The rate of cryptosporidiosis in 0–4 year old was also calculated. Fig. 2 shows the laboratory service areas and associated cryptosporidiosis rates in 0–4 year old between March and June 2000 and is one of 16 such maps that could have been produced for this age group.

### Explanatory variables

For each laboratory service area a number of variables to potentially explain the cryptosporidiosis rates were derived. Living in a rural as opposed to an urban area may increase the probability of contact with livestock. The proportion of the service area in each of eight rural/urban area categories was derived using information from the rural and urban area classification (Bibby and Shepherd, 2001). This dataset groups census areas into four categories (urban, town and fringe, village and dispersed settlements) based upon housing densities. These four classifications are then contextualised further into sparse and less-sparse based upon the housing density at a wider geographical scale.

The social characteristics of an area can affect the behaviour of the population (e.g. individuals of a higher socio-economic status may be more likely to go swimming and walking) which may alter the disease risk. For each service area the percentage of people in each of the eight socio-economic status bands were identified using data from the 1991 and 2001 UK census. Additionally, the proportion of the population aged 0–4 and the percentage employed in agriculture were obtained.

The more agricultural sources of *Cryptosporidium* in an area the greater the probability that an individual will come into direct or indirect contact with *Cryptosporidium*. For each year and month between 1989 and 2003 a 1 km^2^ map of manure applications across England and Wales was obtained from ADAS Consulting. This was created by combining information from the agricultural census with land use data and information from animal excreta and manure management surveys (Anthony et al., 2001). These data were converted into *Cryptosporidium* estimates by multiplying the manure quantities with estimates of *Cryptosporidium* concentrations in the manure taking into account the type of animals and their ages (Hutchison et al., 2004). For each laboratory service area the overlay facilities of the GIS were used to extract estimates of the total amount of *Cryptosporidium* applied to land through animal manures.

Certain drinking water supplies are a risk factor for cryptosporidiosis. Information on water supplies in England and Wales are collected for areas known as water supply zones (WSZs), which are areas of up to 50,000 households receiving identical water supplies. These zone outlines do not coincide with census boundaries. For each laboratory service area the WSZs within were identified using the GIS. Where WSZ outlines crossed service area boundaries the data from the WSZ were allocated to the service area depending upon the proportion of the WSZ population within each service area. WSZs are supplied with drinking water from multiple water treatment works which obtain their water from numerous abstraction points and are subject to different forms of water treatment. Consequently a number of volume weighted measures were derived to describe the water supply of each service area. Information on the proportion of water supplied from different sources (surface vs. groundwater) and subject to different treatments (e.g. membrane filtration, simple disinfection) was obtained from the England and Wales Drinking Water Inspectorate. For each surface or groundwater abstraction, catchments were calculated using the GIS and the density of *Cryptosporidium* applications to land in each were calculated using the 1 km^2^ map of *Cryptosporidium* applications. Information could only be obtained for public water supplies.

Secondary transmission of cryptosporidiosis from imported travel cases may occur. Therefore, within each service area and for each time period the numbers of cases reporting recent foreign travel (excluded from the dependent variable) were obtained.

### Dealing with ascertainment

The cryptosporidiosis data for each laboratory service area may be affected by ascertainment bias which may vary geographically. Several methods were used to examine the impact of this.

Ascertainment of cryptosporidiosis cases is likely to be more complete in younger individuals for two reasons. Firstly different laboratories have different policies for choosing which stool samples to analyse for *Cryptosporidium*, and only testing samples from individuals under 4 years of age is common (Chalmers et al., 2002). Secondly, young children are more likely to be brought to doctors when they are ill (Berkanovic and Telesky, 1985). To examine the impact of this we compared the results of a model using cryptosporidiosis data from all age groups to one using only data from 0 to 4 year olds. Statistically this may reduce the power of the analysis due to lower numbers of cases. However, cryptosporidiosis incidence is higher in young individuals and so selecting this age band only excludes 65% of cases.

In addition to focussing upon cryptosporidiosis in the 0–4 s a further method was developed to account for under-ascertainment of older individuals caused by different laboratory protocols. This involved the creation of a “protocol” variable estimating whether the laboratory is screening all samples for *Cryptosporidium*. This was created using the age profiles of cryptosporidiosis cases from nine laboratories known to be screening all samples for *Cryptosporidium*. These were spread across England and Wales. Using these data the proportion of cases in four age groups (0–4 years, 5–14 years, 15–44 years and >45 years) was determined between 2000 and 2003 for each of the nine laboratories. A mean age structure and standard deviation for this group was then calculated. The age profiles of the other laboratories were then compared to this standard age profile for three time periods (1989–1994, 1995–1999 and 2000–2003) and any with a standard deviation of <1 from the standard age profile were assumed to be screening all samples for *Cryptosporidium*. This produced a new binary explanatory variable “protocol” containing our estimate of whether the laboratory was screening all samples for *Cryptosporidium*. Three time periods were necessary because laboratory protocols may change over time. This analysis estimated that 59% (1989–1994), 68% (1995–1999) and 73% (2000–2003) of laboratories screened all samples for *Cryptosporidium*. These data corroborate the results from previous studies (Chalmers et al., 2002).

A final source of ascertainment bias in IID surveillance data is the under-reporting of cases reporting recent foreign travel. Although foreign travel cases were removed from our analysis it is therefore likely that many of these cases remained. Consequently, we created a variable indicating the number of cryptosporidiosis cases reporting recent foreign travel within each laboratory service area as a marker of secondary transmission. This variable may be correlated with unreported foreign travel cases and will therefore act as an indicator of this ascertainment bias. In the 0–4 year old model this variable was the 0–4 year rate of foreign travel cases. In the all age SIR model this was the all age SIR of foreign travel cases.

### Statistical modelling

To account for the continually changing service area configuration, and to investigate whether the factors underlying cryptosporidiosis geography vary seasonally, cryptosporidiosis data were extracted for each laboratory service area for three seasons for every year between 1989 and 2003. The seasons were March–June, July–October and November–February. These correspond to the spring peak in incidence, the late summer and autumn peak, and the low incidence over the winter months, respectively (Lake et al., 2005).

To explore the factors affecting these geographical patterns, multivariate models were produced to examine the impact of the explanatory variables upon cryptosporidiosis in each of the three seasons. All analysis was undertaken in StataSE 8.0. The models were produced using a random effects model which modifies the standard linear predictor to include an amount that varies randomly between years (Kirkwood and Sterne, 2003). A forward entry regression technique was performed by adding the most significant variable in turn. Collinearity was avoided by ensuring that the addition of each variable did not lead to significant changes in the coefficients or significance of any other variables in the model. Standardised forms of the independent variables were entered into the models. Results are presented in terms of coefficients and significance (*p*-values).

For each season a model using the 0–4 cryptosporidiosis rate as the dependent variable was produced. This outcome variable was chosen because it is least likely to be affected by ascertainment bias. The March–June and the July–October periods produced models with identical explanatory variables. The November–February model included a subset of these explanatory variables. To allow comparison between seasons identical explanatory variables were consequently included in each model.

To ensure that the results were not driven by regional differences in incidence, each was tested by including a set of regional dummy variables. The inclusion of these did not have a significant impact on any of the results. In addition all models were fitted with and without significant outliers. In nearly all cases, the removal of outliers strengthened the relationships presented.

A number of alternative models were produced to examine the impact of ascertainment bias upon the results. This bias is likely to be greater in the all age SIR. Consequently, a new model was produced using an identical set of explanatory variables but substituting the 0–4 cryptosporidiosis rate with the all age SIR as the dependent variable. Both the 0–4 year old model and the all age SIR model will produce similar results if the ascertainment bias in the all age SIR is uncorrelated with our explanatory variables.

To examine whether the ascertainment bias introduced by different laboratory protocols has an impact upon the results, the model with the all age SIR as the dependent variable was run with and without the variable indicating our estimate of laboratory protocol. If laboratory under-ascertainment is uncorrelated with our explanatory variables we would expect models with and without this variable to be similar.

Foreign travel is under-reported in surveillance datasets. Consequently, the model with the 0–4 cryptosporidiosis rate as the dependent variable and the model using the all age SIR (including the protocol variable) were run with and without the variables indicating the amount of reported foreign travel in these age groups. The models with and without the travel variables were then compared to examine whether significant changes in the model occurred.

## Results

The results of the statistical analysis are presented in Table 1. If we consider the three initial models for the 0–4 age group (one for each season and without the travel variable), then these indicate that cryptosporidiosis incidence is higher in rural laboratory service areas but not during the winter period. There was also a positive association between cryptosporidiosis incidence and the density of *Cryptosporidium* from animal manures in the service area for all time periods. Finally, cryptosporidiosis is positively associated with laboratory service areas supplied by poorer water treatment in all time periods. Variables indicating the proportion of people in each of the eight socio-economic status bands, the percentage of the population aged 0–4 and the proportion employed in agriculture were not significant.

Several methods were applied to examine the impact of ascertainment upon the results. The first was to compare the results from the 0–4 age group, the dependent variable with which we have most confidence, to that of the all age SIR. Table 1 demonstrates that both models produced similar results in terms of the significant explanatory variables. However, the 0–4 age group model had similar or higher explanatory powers than the all age SIR. In addition, this model produced consistently stronger (more significant) relationships with the explanatory variables.

The second method was to include the protocol variable as an independent variable. This is our estimate of whether the laboratory was screening the samples from all age groups for *Cryptosporidium*. When this was added to the all age SIR model the protocol variable was significant or nearly significant in all three periods. Therefore, this variable is successfully identifying laboratory service areas with higher cryptosporidiosis incidence. However, although significant this variable has a small impact upon the explanatory power of the models and the strength of the other explanatory variables.

The final method to examine the impact of ascertainment was the inclusion of a variable indicating the quantity of reported foreign travel cases in the laboratory service area. Table 1 demonstrates that in five of the six models this variable was statistically significant. The most striking impact of the travel variable is that it consistently enhanced the significance of the other explanatory variables especially in the all age SIR model. The travel variable nearly quadrupled the explanatory power of the all age SIR model in the July–October period, highlighting the importance of imported foreign travel cases during this period.

## Discussion

One limitation of cryptosporidiosis and other IID surveillance data in England and Wales and other countries, is that case location is frequently only known in terms of the laboratory to which the sample was sent for analysis. This paper has presented methods through which such data can be converted into area-based rates. By producing health data which are multiples of census units it has demonstrated how these data can be linked to potential explanatory variables such as land use information and the socio-economic status of the area in a straightforward manner. It has also shown how IID surveillance data can be linked to water supply information.

The validity of these methods is enhanced by the observation that the associations between cryptosporidiosis and the explanatory variables are consistent with previous research. Poor water treatment is a well known risk factor for cryptosporidiosis (Lisle and Rose, 1995). Living in an area with a large density of *Cryptosporidium* in manure is associated with elevated cryptosporidiosis incidence. This may be due to the increased probability of direct contact with the environmental reservoir of *Cryptosporidium*. Cryptosporidiosis incidence was also higher in more rural service areas suggesting that individuals living in these locations are more likely to interact with their surroundings increasing the probability of *Cryptosporidium* infection. This impact is significant event when the amount of *Cryptosporidium* in the service area has been controlled for. However, this factor is insignificant between November and February. This would support the hypothesis of rural lifestyles, as in the winter months individuals are less likely to interact with their surroundings. Finally, the variable indicating the amount of cases reporting recent foreign travel in the laboratory service area was positively associated with cryptosporidiosis. This supports the hypothesis that many travel cases are unreported in national surveillance. Although this variable was predominantly used as a check for ascertainment bias, it may also indicate secondary transmission from foreign travel cases. In such an analysis it is not possible to disentangle these factors.

This study was only able to compare cryptosporidiosis incidence between 136 different geographical areas. Each contained approximately 350,000 individuals and covered an average area of 1142 km^2^. Such large areas will blur social and environmental heterogeneities within each service area, making it more difficult to explore the factors underlying disease geography. In spite of such limitations this paper demonstrated that these areas are still useful in understanding disease geography. The occurrence of more precise geographical information (e.g. postcodes) on national IID surveillance data is increasing and so in the future it may be possible to undertake similar studies for finer geographical units.

One important issue in the use of IID surveillance data to explore the spatial patterns of disease is ascertainment bias and whether this affects the results of the analysis. This paper explored this problem in detail. Consistent results were presented between models predicted to be least (0–4 rate) and most (all age SIR) affected by ascertainment bias. Furthermore, the results were stable when variables related to ascertainment (laboratory protocol and reported foreign travel) were included in the model. This suggests that, in spite of known ascertainment bias in IID surveillance datasets, in the case of cryptosporidiosis at least, these data are useful for examining the factors underlying disease geography. Variations in ascertainment do not appear to be correlated with the variables of interest. When ascertainment bias was examined in the analyses (i.e. comparing results between the 0–4 age group model and the all age SIR model, including a laboratory protocol variable in the analysis, and incorporating the number of reported foreign travel cases) in all instances the resultant models appeared stronger. This was most pronounced when the quantity of reported foreign travel cases was included in the models. Such approaches are therefore recommended in future studies.

There are two main ways of dealing with ascertainment bias; prevention or estimation, and correction (Sethi et al., 1999). In this paper we have successfully demonstrated methods to take account of this artefact in an analysis. However, such techniques are an imperfect substitute for preventing ascertainment bias. Several important elements affecting ascertainment bias have been discussed in this paper and we encourage public health officials to explore how these artefacts may be minimised. This paper re-emphasises that more research is needed into the bias and confounding that ascertainment may introduce into IID surveillance data.

## Conclusions

This paper has presented a suite of techniques demonstrating how IID surveillance data can be converted into area-based illness rates and linked to datasets of possible explanatory variables. The case study of cryptosporidiosis presented demonstrated that illness rates were higher in rural areas, localities with more agricultural manure and areas with poorly treated water supplies. These are consistent with previous research into cryptosporidiosis validating the methods used. Although ascertainment bias is a persistent concern in the use of IID surveillance data, this paper has demonstrated methods through which the severity of this can be explored. These indicated that ascertainment bias is unlikely to confound an analysis into the factors underlying disease geography. Worldwide, IID surveillance data are an importance resource to study disease geography. This paper demonstrates that, using appropriate methods, they can provide useful clues into the factors underlying disease aetiology.

## Figures and Tables

**Table 1:** Random effects model for cryptosporidiosis in laboratory catchments between 1989 and 2003^a^

Cryptosporidiosis dependent variable	Independent variables					
	Standardised proportion of rural area in laboratory service area	Standardised quantity of *cryptosporidium* in laboratory service area	Standardised proportion of poor water treatment	Standardised protocol	Standardised foreign travel variable	*R*^2^ overall %
March–June						
0–4 year old rate	0.3106 (0.002)	0.7366 (0.000)	0.2183 (0.020)	–	–	14.57
0–4 year old rate^b^	0.3230 (0.001)	0.7189 (0.000)	0.2076 (0.021)	–	0.1636 (0.000)	15.85
All age SIR	0.1476 (0.167)	0.6730 (0.000)	0.1713 (0.096)	–	–	10.27
All age SIR^c^	0.1502 (0.153)	0.6567 (0.000)	0.1754 (0.084)	0.2101 (0.046)	–	10.96
All age SIR^d^	0.1924 (0.043)	0.6032 (0.000)	0.1670 (0.067)	0.1069 (0.022)	0.2733 (0.000)	15.27
July–October						
0–4 year old rate	0.1769 (0.074)	0.3254 (0.000)	0.2100 (0.034)	–	–	5.18
0–4 year old rate^b^	0.1946 (0.035)	0.3244 (0.000)	0.2041 (0.026)	–	0.2144 (0.000)	8.04
All age SIR	0.0832 (0.442)	0.2232 (0.025)	0.1633 (0.132)	–	–	3.43
All age SIR^c^	0.0857 (0.422)	0.2118 (0.032)	0.1627 (0.127)	0.1910 (0.068)	–	4.48
All age SIR^d^	0.1362 (0.140)	0.2023 (0.020	0.1490 (0.105)	0.1152 (0.012)	0.4137 (0.000)	15.09
November–February						
0–4 year old rate	0.1003 (0.276)	0.3645 (0.000)	0.2449 (0.007)	–	–	5.51
0–4 year old rate^b^	0.0994 (0.260)	0.3643 (0.000)	0.2418 (0.005)	–	0.3249 (0.381)	5.71
All age SIR	0.0109 (0.916)	0.3137 (0.002)	0.2545 (0.013)	–	–	5.45
All age SIR^c^	0.0152 (0.884)	0.3012 (0.002)	0.2194 (0.034)	0.2194 (0.034)	–	6.21
All age SIR^d^	0.0317 (0.736)	0.2894 (0.001)	0.2301 (0.013)	0.1182 (0.021)	0.2648 (0.000)	11.19

a Figures in parentheses represent *p*-values.

b Model includes the foreign travel variable as an independent variable.

c Model includes the protocol variable as an independent variable.

d Model includes the protocol and foreign travel variables as independent variables.
